# Editorial: Athlete psychological resilience and digital mental health implementation

**DOI:** 10.3389/fpsyg.2022.1082998

**Published:** 2022-11-29

**Authors:** Luke Balcombe, Diego De Leo, Martin J. Turner

**Affiliations:** ^1^Australian Institute for Suicide Research and Prevention, School of Applied Psychology, Griffith University, Brisbane, QLD, Australia; ^2^Department of Psychology, Faculty of Health and Education, Manchester Metropolitan University, Manchester, United Kingdom

**Keywords:** psychological resilience, athlete, digital mental health implementation, stress, coping

## Introduction

An athlete's ability to adapt to stress and adversity is vital for their psychological resilience. While resilience has been traditionally considered from a physiological perspective in sports, in recent years, the importance of the mind-body connection has led to a growing interest in the field of athlete psychological resilience.

In this Research Topic, we have received papers evaluating different approaches to psychological resilience in elite athletes, endurance athletes (ranging from competitive amateurs to professional athletes), and tactical athletes also known as high-performance military personnel. Three of the four articles of this collection primarily examined athlete psychological resilience and one article also focused on digital mental health implementation. Each article is presented separately because of the investigation of different types of athletes in addition to the exploration of cognitive and/or psychological resilience.

## Cognitive resilience to psychological stress in military personnel

The existing literature on cognitive resilience showed how cognitive functions oppose the effects of stress (Staal et al., 2008). The individualized experience of psychological stress is related to the perceived or anticipated stressor (Roesch et al., 2002) and mediated by cognitive appraisal, coping, and reappraisal (Lazarus and Folkman, 1984). For example, the impact of physical and mental training as well as competition in athletes emphasizes the need for developing and sustaining effective physiological and cognitive functioning skills. Tactical athletes are expected to operationalize a specific role. In addition to physical variations to achieve operational performance, the high prevalence and significant consequences of intense psychological stress in athletes indicate that understanding cognitive resilience is essential for appraising and coping with stressors inside and outside of their occupation.

Flood and Keegan highlighted the complexity of underlying psychological stress experienced by military personnel in a review of the situations where cognitive resilience is challenged. Findings suggest that military personnel experience similar common occupational stressors as civilian populations in addition to potentially traumatizing combat stressors related to injury and death. However, more work is needed to assess these combat stressors or potentially traumatizing events through a validated measure of appraisal and coping in addition to a qualitative interview to clarify that they are appraised as stressful. The results have shown that the psychological stress of modern warfare has exacerbated the impact on cognitive performance. For example, the “persistent conflict” state marked by the unpredictability of insurgent attacks and the increased use of technology such as operating unmanned aerial vehicles (UAVs) justifies provoking stress in an ecologically valid way. Virtual Reality (VR) exercises may help prepare combatants to be adaptive in their cognitive functioning when experiencing the subjective accumulation of stress.

The beneficial effect of cognitive resilience is reinforced by existing theoretical models about mitigating the effects of stress on cognitive performance through effortful allocation and reallocation of attention to achieve task effectiveness. The integration of these models into military settings in addition to the detection of decreased processing efficiency was proposed by Flood and Keegan to contribute to a better understanding of cognitive resilience to psychological stress. Consequently, it was suggested to extrapolate the subjective experience of stress and its impact on the performance of cognitive operations through the broader use of self-report measures and mixed methods approaches to enhance cognitive resilience. Additionally, it was recommended to use tailored technology for examining the evolving environment in which tactical athletes operate.

## An app-enhanced cognitive fitness training program for athletes

The existing literature on using technology to improve fitness is focused on translating physiological biofeedback signals into meaningful and actionable insights. The next frontier for utilizing technology in sports is to explore brainwave patterns from training cognitive fitness to enhance both physical and mental performance in addition to wellbeing and better results in competition.

Aidman et al. introduced digital mental health implementation to operational performance in elite athletes through the development of a cognitive fitness training smartphone app. The Cognitive Gym program applies the Cognitive Fitness Framework (CF2) which is based on the Research Domain Criteria (RDoC) Framework. The app aims to identify the cognitive processes underlying normal and abnormal functioning to improve psychological resilience and reduce stress. The prototype involves 30 min of daily practice with the app for 3 weeks. Training drills address the domains of confidence, self-belief, and a mastery vs. outcome focus. The training is primarily delivered through educational videos and reading adapted from the CF2 model. The app includes guided cognitive workouts, breathing sessions, user engagement tools, completion trackers, leaderboards, and a social feed. A pre- and post-training evaluation is conducted by a machine learning algorithm in addition to the users' and coaches' evaluation of the users' performance.

Although qualitative feedback found there is the promise of efficacy and user acceptance, the expectation is that the use of the Cognitive Gym app and its supporting materials will lead to better overall cognitive fitness and wellbeing because it is (1) based on the CF2 model (2) targets constructs throughout the performance cycle (3) uses drills that passed rigorous testing (4) applies a comprehensive psycho-educational package and (5) provides the core sequence of the prototype for external evaluation. The athlete-focus of the Cognitive Gym app may counter COVID-19 disruptions to sports industry support programs by providing self-guided, gold-standard training for mental capacities, mental readiness, and adjustment skills to assist athlete psychological resilience in competition.

## Coping and resilience among endurance athletes during COVID-19

The existing literature on the effect of pandemics on psychological wellbeing focused on stress and social isolation which can negatively impact mental and cardiovascular health. Similar findings were reported for COVID-19 lockdowns which invoked increased stress (Di Fronso et al., 2020), reduced physical activity (Ruiz et al., 2021), and decreased wellbeing (Lades et al., 2020), as well as an excess of negative psychological outcomes: primarily anxiety, depression, and stress (Chtourou et al., 2020). Although the worst of COVID-19 and associated lockdowns appear to be in the past, the threat of future pandemic disruptions provides the context for obtaining insights into athletes' resilience and general coping strategies. It is relevant because athletes were constrained in their training opportunities resulting in physical and mental adversity. Resilience theory is well-established in the athlete population marked by appraisal before emotional and coping responses leading to a positive, protective impact. Coping theory was described as a response to stress with differing effectiveness in resolving significant issues.

Harman et al. investigated the extent that endurance athletes exercised during lockdown to understand how they enacted dispositional resilience and coped with subjectively perceived barriers to training. The analyses indicated that endurance athletes who exhibited greater athletic levels also exhibited greater lockdown resilience and adaptive cognitive-emotional coping strategies in addition to perceived lower barriers to training during lockdown. The experience of lockdown hardship depends on the level of the athlete, with elite athletes having more contextual adversity from disrupted training plans than amateur athletes.

Overall, the mixed methods study supports previous findings on psychological resilience that it is a personal asset critical to promoting functional adaptation to the potential negative effect of stressors. In this case, endurance athletes were found to have been likely to overcome unfavorable lockdown conditions. The cross-sectional study also supports the previous claim that the resilience of elite athletes depends on the accessibility of resources and the context. However, COVID-19 was noted as a particularly unique disruption in terms of the barriers to training resources and psychological support. Remarkably, the study applied the commonly used Connor-Davidson Resilience Scale (CD-RISC-25) which focuses on an individual's ability to regain biopsychological balance through maintaining goal focus in challenging circumstances. It was recognized as a limitation that there is a need for a sporting resilience measure that integrates athlete-specific mental health considerations in a biopsychosocial approach. Subsequently, it was recommended that future research extend into a longitudinal design to capture the factors internal and external to sport that influence resilience.

## The sporting resilience model

Psychological resilience has grown incrementally in the past two decades and is broadly used as a common language to efficiently thwart socio-cultural differences. The disruptive impact of COVID-19 changed the structure of sport, making sporting resilience of even more consequence. The existing literature on athlete psychological resilience emphasized how athletes are confronted by adversity and intense experiences that contribute to their unique stressors and mental health issues. However, narrative reviews preceded the recent increase in publications in the sporting domain.

Gupta and McCarthy conducted a systematic review of resilience research in sport and exercise psychology with theoretical considerations also from positive and clinical psychology to provide an up-to-date summary of the evidence base and future directions for research. The integrative and inductive review found that the foundational definition of athlete psychological resilience required updating to improve empirical precision specific to the sporting domain. For example, the operationalization of resilience to date is comprised of different components including the influence of environmental and sociocultural contexts inside and outside of sport in addition to the maintenance of positive equilibrium in one's biopsychosocial system when confronted by multiple challenges. The review found a need to go beyond protection from stressors/adversities to also encompass positive adaptation over time including rebounding when new challenging situations arise.

Fundamentally, the existing theory on dynamic process-trajectory underpins sporting resilience whereby performance and adaptation capacity increase while sustainably engaging with protective resources. The collation of empirically supported sporting resilience components was synthesized into a distinct process involving dynamic person-environment-adversity interaction. The new definition centered on an athlete's ability to assess their experience in the face of adversity which lets them perform in line with their existing level and continue adapting beyond that. The Sporting Resilience Meta-Model presented a new theory suggesting that a resilience filter comprised of biopsychosocial protective factors controls the impact of adversity and determines the course of positive adaptation. However, the protective factors differ between individuals and environments and may change over time. Thus, the new definition of sporting resilience proposed a quite stable notion. The flexible list of protective factors may be tested and adjusted to reflect future interaction and dynamicity as well as a resource for mapping the trajectory of an athlete's resilient adaptation.

## Conclusions

The Research Topic is timely and topical after COVID-19-related sporting disruptions lifted interest in athlete psychological resilience as an important concept for achieving optimal performance in the face of stress and adversity. Primarily, the systematic review synthesized the evidence base into a new theory that typifies how resilient athletes overcome various challenges to procedurally adapt to adversity over a course of time. This was integrated into a testable model based on the individualized experience of filtering biopsychosocial protective factors inclusive of environmental and sociocultural influences inside and outside of sport.

This collection's three other articles discuss innovative cohort engagement. A review of cognitive resilience in military personnel found the need for tactical training for efficient and effective high performance in operationalizing warfare tasks (e.g., applying VR training to prepare against the threat of UAVs). The promise of tailored digital solutions is also exemplified by the Cognitive Gym app protocol which describes a program that contributes to self-guided training in elite athletes to increase their cognitive capacity to be psychologically resilient in competition. The research findings with endurance athletes revealed the importance of the context in which athletes train and compete—the more that was at stake from barriers and adverse disruptions, the more resilience was exhibited.

Overall, the articles point toward the next steps such as implementing mixed methods in longitudinal studies to grow the evidence base and evaluating the new model with international comparisons. Future studies are recommended to (1) build upon the evidence-based foundations for athlete psychological resilience; (2) integrate technology in high performance-resilience interventions; (3) test and provide an outcome measure for mapping the trajectory of athlete psychological resilience; and (4) advance a model of delivery for effectively measuring athlete psychological resilience.

## Author contributions

LB contributed to the writing. DD and MT contributed to the review. All authors have made a direct and intellectual contribution to the work and approved it for publication.

## Conflict of interest

The authors declare that the research was conducted in the absence of any commercial or financial relationships that could be construed as a potential conflict of interest.

## Publisher's note

All claims expressed in this article are solely those of the authors and do not necessarily represent those of their affiliated organizations, or those of the publisher, the editors and the reviewers. Any product that may be evaluated in this article, or claim that may be made by its manufacturer, is not guaranteed or endorsed by the publisher.
